# Computer Simulation Insight into the Adsorption and Diffusion of Polyelectrolytes on Oppositely Charged Surface

**DOI:** 10.3390/polym15132845

**Published:** 2023-06-28

**Authors:** Anna A. Glagoleva, Alexander A. Yaroslavov, Valentina V. Vasilevskaya

**Affiliations:** 1 A. N. Nesmeyanov Institute of Organoelement Compounds, Russian Academy of Sciences, Moscow 119991, Russia; starostina@polly.phys.msu.ru; 2 Department of Chemistry, M.V. Lomonosov Moscow State University, 1-3 Leninskie Gory, Moscow 119991, Russia; yaroslav@belozersky.msu.ru

**Keywords:** polyelectrolytes, adsorption, diffusion, surface coatings, computer simulation

## Abstract

In the present work, by means of computer simulation, we studied the adsorption and diffusion of polyelectrolyte macromolecules on oppositely charged surfaces. We considered the surface coverage and the charge of the adsorbed layer depending on the ionization degree of the macromolecules and the charge of the surface and carried out a computer experiment on the polymer diffusion within the adsorbed layers, taking into account its strong dependency on the surface coverage and the macromolecular ionization degree. The different regimes were distinguished that provided maximal mobility of the polymer chains along with a high number of charged groups in the layer, which could be beneficial for the development of the functional coatings. The results were compared with those of previous experiments on the adsorption of polyelectrolyte layers that may be applied as biocidal renewable coatings that can reversibly desorb from the surface.

## 1. Introduction

Polyelectrolyte adsorption on an oppositely charged surface from an aqueous solution plays a crucial role in various biological phenomena and is an important factor in numerous practical applications. Among them are the development of functional coatings and thin films, soil and water treatment, drug delivery, heterogeneous catalysis, chemical sensing, lubrication, adhesion, etc. [1,2,3,4].

The development of such systems includes the consideration of many factors that determine the conformations of the adsorbed macromolecules and the structure of the adsorbed layer as well as the diffusion processes within it [5,6,7,8]. Obviously, all these phenomena depend primarily on the concentration of the macromolecules on the surface, i.e., the polymer surface coverage.

At a low polymer surface coverage, the intermolecular interactions are weak; macromolecules can spread along the surface and obtain the optimal conformation, called “de Gennes pancake” [5]. In this case, chains are able to adsorb the maximal number of units on the surface. In the dilute limit, theory predicts the Rouse model scaling dependence of the diffusion coefficient *D* of their centers of mass on the number of chain segments *N*: *D* ~ *N*^−1^ [5]. Experimentally, such dependence is observed for very smooth surfaces [9]; in other cases *D* ~ *N*^−3/2^, it is consistent with two-dimensional diffusion between the obstacles [4,9,10]. In a two-dimensional monolayer with densely packed chains that do not entangle with each other, macromolecule diffusion is ameboid-like, with *D* ~ *N*^−5/8^ [11,12,13,14]. This law is derived from the number of contacts of an adsorbed chain with the surrounding chains in the two-dimensional melt.

An increase in the polymer surface coverage leads to a decrease in the adsorbed area per polymer chain. First, macromolecules that are still being adsorbed shrink in the direction along the surface; then, with a further increase, macromolecules form loops and tails and then overlap and entangle. In such layers, the macromolecules diffuse by a reptational mechanism. Their diffusion coefficient is estimated by the ratio of the terminal reptation time τ_rept_ ~ *N*^3^ and the gyration radius in a good solvent in the two-dimensional case *D* ~ *R*^2^_g_/τ_rept_ ~ *N*^−3/2^.

Accordingly, three diffusion regimes have been observed in experiments [15,16] on the growth of the polymer surface coverage, as described below. The lateral diffusion coefficient *D* first increases, presumably because the number of adsorption sites per molecule decreases as chains switch from a flatly adsorbed to loop-train-tail conformation. When the overlap concentration is reached, *D* decreases, reflecting the crowding and entanglement of the chains. At the highest coverage, diffusion becomes faster as a result of weak interactions of the adsorbing chains with a formed homogeneous brush layer on the surface. According to these results, the diffusion coefficient is a non-trivial function of the polymer surface coverage.

Upon the adsorption of the polyelectrolyte chains on an oppositely charged surface, the polymer surface coverage is, to a large extent, determined by the surface charge density. First, the polymer surface coverage monotonically grows with the increase in the surface charge, and then, as full saturation is approached, it reaches a plateau. The total charge of the adsorbed polymer can be either equal to or higher than the total charge of the surface it is adsorbed on. In the latter case, this phenomenon is called overcharging [17]. The overcharging phenomenon is explained by the detaching of some polyelectrolyte chain segments from the charged surface in favor of placing more chains on that surface [18]. It strongly depends on the macromolecule’s degree of ionization and is influenced by solvent-mediated polymer–polymer and polymer–surface interactions. The tendency for lower ionization degrees of polyelectrolytes to facilitate the adsorption of a larger amount of polymer has been demonstrated in a number of simulations and experimental works [19,20,21].

The non-electrostatic interactions (solvent quality) are especially important in the investigation of weakly charged polyelectrolytes, where electrostatic and non-electrostatic interactions between the monomer units are comparable [19,20,21,22,23,24]. Such macromolecules have only some of their monomer units adsorbed on the surface and can form many loops, even at a low polymer surface coverage. They can non-monotonically change their size upon worsening solvent quality; they first spread out on the surface and then collapse into drops [25]. The diffusion coefficients of such macromolecules are non-monotonic functions of the surface charge density [25].

The influence of the surface charge density and surface charge distribution, the strength of the polymer–surface and solvent-mediated polymer–polymer interactions on the polymer surface coverage, and the structure of the adsorbed layer were studied in [20] by means of a molecular dynamics simulation. Particularly, the growth of the surface coverage with an increase in the surface charge density was observed in all cases except for the case of a hydrophilic polymer on the hydrophilic surface, where the saturation of the surface coverage was achieved at a high surface charge density, thus precluding the surface overcharging.

By means of various experimental techniques, it has been shown that polymer–surface interactions influence the mechanism of diffusion. It can be predominantly two-dimensional with a strong attraction between the polymer and the surface, or it can be consistent with three-dimensional biased Brownian motion containing desorption hops, subsequent multiple surface encounters before readsorption, and in-plane 2D diffusion during the waiting times between the hops [26,27,28,29].

In the present study, we investigated the effect of the surface charge and the ionization degree of the macromolecules on the process of their adsorption, the structure of the adsorbed layer, the surface coverage, and the process of their in-plane diffusion. For the first time, we considered the simultaneous influence of two opposing factors acting with an increase in charge carried by each macromolecule—a decrease in the surface coverage, which favors diffusion, and an increase in the total layer charge, which hinders diffusion.

The computer simulation studies were supported by the analysis of the previous experimental work.

## 2. Model

To solve the tasks set out in the article, we have considered a solution of polyelectrolyte chains in the vicinity of the uniformly charged flat surface, simulated their adsorption, and then studied the structure of the adsorbed layer and diffusion of the adsorbed macromolecules. The charged surface was represented as a smooth plane with the smeared negative charge deposited on it with the surface density σ_surf_.

**Figure 1:**
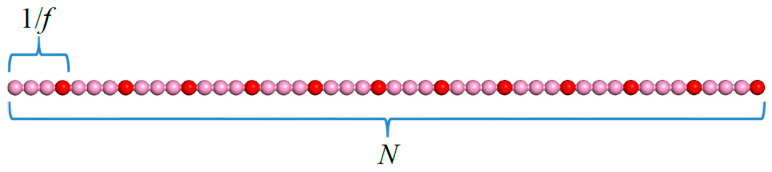
Schematic representation of a macromolecule; dark beads represent charged units.

The solution contained *M* macromolecules of *N* linearly connected monomer units each. Positively charged groups were distributed uniformly along the polymer chains, and the fraction of charged monomer units (ionization degree) varied from *f* = 0.0625 to *f* = 1.0 (Figure 1). The electrical neutrality of the simulation cell was ensured by *f* × *M* × *N* counterions compensating for the charge of the macromolecules.

The chains of the polyelectrolyte were presented in a framework of a bead-spring model. The excluded volume of the polymer groups and counterions was accounted for by the repulsive part of the Lennard-Jones potential:

(1) $$E_{LJ}\left(r_{ij}\right)=\left\{\begin{array}{c} 4\varepsilon \left[{\left(\frac{\sigma }{r_{ij}}\right)}^{12}-{\left(\frac{\sigma }{r_{ij}}\right)}^{6}+\frac{1}{4}\right]\\ 0,r_{ij}>r_{c} \end{array}\right.,r_{ij}\leq r_{c},$$

where $r_{c}=\sqrt[{6}]{2}$ is the cutoff radius (and the radius of a monomer unit or a counterion). The parameters ε=1 and σ=1 are responsible for energetic and spatial scales, respectively, and the simulation results are presented in terms of these quantities.

The beads comprising polymer chains were connected by applying the rigid spring potential:

(2) $$E_{bond}\left(r_{ij}\right)=K{\left(r_{ij}-b\right)}^{2},$$

where *K* = 10,000 ε/σ^2^ is the coefficient, $r_{ij}$ is the distance between *i*-th and *j*-th units, and the bond length *b* = 1 is kept fixed with its variation limited to 1%.

Charged groups of the polyelectrolyte and counterions interacted via the Coulomb potential:

(3) $$E_{El}\left(r_{ij}\right)=k_{B}Tl_{B}\frac{q_{i}q_{j}}{r_{ij}},$$

where *k_B_T* = ε = 1 and *l_B_* = 4 is the Bjerrum length—the distance where the electrostatic interaction between two charged groups becomes equal to *k_B_T*. The charged groups bear unit charges, *q_i_* = −1 or +1. The Ewald summation method was used for calculations of electrostatic interactions [30], treating the interaction potential as a sum of two terms, the short-range contribution calculated in a real space and a long-range contribution calculated in a reciprocal space using the Fourier transform.

The chains and counterions were placed into a rectangular prism simulation cell with periodic boundary conditions along the *x* and *y* directions and non-periodic along the *z* direction. The verges of the cell in the *z* direction were the impenetrable walls interacting with all the beads via (9-3) the Lennard-Jones potential:

(4) $$E_{w}\left(r_{ij}\right)=\left\{\begin{array}{c} \varepsilon \left[{\frac{2}{15}\left(\frac{\sigma }{r_{iw}}\right)}^{9}-{\left(\frac{\sigma }{r_{iw}}\right)}^{3}\right]\\ 0,r_{iw}>r_{cw} \end{array}\right.,r_{iw}\leq r_{cw},$$

where $r_{iw}$ is the distance between the bead and the wall; $r_{cw}=2.5$ is the cutoff distance.

The bottom verge of the cell (*z* = −2), perpendicular to the *z*-axis, was considered a uniformly negatively charged plane and its effect was introduced via an electric field acting on the charged monomer units in the direction perpendicular to this plane [25,31,32].

Thus, an additional constant F*_z_ force was applied in the *z*-axis direction so that the plane attracts positively charged beads and repulses negatively charged ones. This force is directly connected with the Bjerrum length and surface charge density σ_surf_:

F*_z_ =−2π*l_B_*σ_surf_*q_i_*. (5)

The surface charge σ_surf_ is set implicitly using a special procedure described below and varies in the interval in such a way that the largest Gouy-Chapman length *l*_GC_ was of the order *l*_GC_~3.

The sizes of the cell along the *x* and *y* axes (*L_x_* = *L_y_* = 140) were chosen to be significantly larger than the coil size, as the lateral diffusion crucially depends on a system size [33]. However, *L_x_* = *L_y_* were much smaller than the cell size along the *z*-axis (*L_z_* = 402) to minimize the effect of the periodic replicas in the *z* direction [34] and the effect of the neutral plane. This approach is widely used in simulations of the interfacial systems [35,36,37,38]. It also has an advantage over the simple truncation of electrostatic interactions as the latter results in artificial maxima and minima in radial distribution functions at the cutoff distance [35,39]. Moreover, the slab Ewald method [40,41,42] was applied by introducing the gap of the height 2*L_z_* in the simulation cell along the non-periodic *z* direction and using a standard 3D Ewald method with the summation order changed to the slabwise one.

In the initial state, the polymer chains were arranged in rows in the bottom part of the simulation cell, while the counterions were distributed randomly within the whole volume of the cell preserving the electroneutrality of the system. The values *M* = 512, *N* = 48; *M* = 384, *N* = 64; *M* = 256, *N* = 96; and *M* = 192, *N* = 128 with the total number of monomer units *M* × *N* = 24576, or *M* = 308, *N* = 80; *M* = 220, *N* = 112 with the total number of monomer units *M* × *N* = 24640 were considered.

The time evolution of the system was computed by means of Langevin dynamics simulations using the LAMMPS program package [43]. Langevin uncorrelated noise term $F_{r}\sim \sqrt{\frac{2k_{B}Tm_{b}}{Ddt}}$ and drag force term $F_{f}=-\frac{m_{b}}{D}v$ were introduced into the equations of motion. Here, $m_{b}=1$ is the mass of each group, v is its velocity, $dt=0.005\sigma \sqrt{\frac{m_{b}}{\varepsilon }}$ is the integration step, and *D* = 100 is the damping factor, specifying that temperature is relaxed within 100 time steps. The temperature was kept at *T* = ε/*k_B_* = 1.

**Figure 2:**
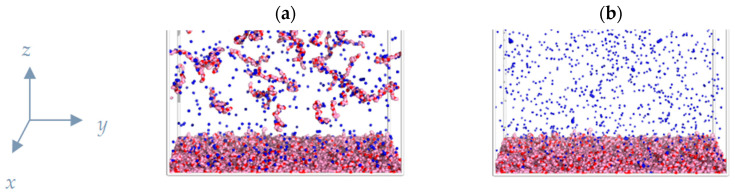
Snapshot of a simulation cell fragment after equilibration from the initial state (**a**). Snapshot of a simulation cell fragment after the “rinsing” step and addition of the counterions (**b**).

The first stage of the molecular dynamics simulation was the equilibration performed for 0.5 × 10^6^ timesteps (Figure 2a) so that the energy standard deviation did not exceed 5%. Then, the “rinsing” stage took place: all the macromolecules which do not have any monomer units within *z* < 1 (within the largest Gouy–Chapman length) and all the counterions were withdrawn from the simulation cell and counterions for neutralizing the macromolecules left in the cell were added (Figure 2b).

After the subsequent short equilibration, the “rinsing” was repeated and the production run of 16 × 10^6^ timesteps was carried out. During subsequent calculations (equilibration and production run), not a single macromolecule leached from the surface.

During the production run, the total charge of the adsorbed layer, the thickness of the layer, the gyration radius, and mean-square displacements along the *xy*-plane (MSD) of the center of mass of each adsorbed macromolecule were calculated. The thickness was estimated as a mean *z*-component of the gyration tensor of the adsorbed macromolecules. The MSD values averaged over all the macromolecules in the cell and over several runs were used to find the lateral diffusion coefficient *D* as the slope in the dependencies of MSD on time (the slope is equal to 4*D*; see the exemplary plot in Figure S1 of the Supplementary Materials).

The influence of the negatively charged counterions collected near the *z* = 400 neutral plane during the simulation was taken into account as a portion of the F*_z_ field (Equation (5)) as they are distributed uniformly along that plane [40,41,44,45]. The resulting surface charge of the *z* = −2 plane was calculated as follows: *Q*_surf_ = *F_z_* × *L_x_* × *L_y_*/(2π × *l_B_*) – *Q*_up_, where *F_z_* is a constant predefined field acting along the *z*-axis and *Q*_up_ is the total charge of the ions concentrated at the upper verge of the simulation box. At the fixed *F_z_*, the value of *Q*_up_ variation was about 1% for different systems, so the resulting surface charge *Q*_surf_ was considered to be constant. The surface charge density was defined as σ_surf_ = *Q*_surf_ × *S*_0_/(*L_x_* × *L_y_*). For simplicity, we operate with the value normalized by the area $S_{0}=2\sqrt{3}r_{c}^{2}$, which is the area that would be occupied by each charge on the surface if the surface was composed of the hexagonally (most densely) packed particles with a radius equal to the radius of a monomer unit $r_{c}=\sqrt[{1/6}]{2}$ (see Equation (1) for the excluded volume of the bead).

In accordance with the aim of the study, we have investigated the surface coverage and the chains conformations of the adsorbed polymer depending on the charge of the surface σ_surf_ and ionization degree of the polycations *f*. The surface coverage Γ was calculated as the total amount of monomer units in the adsorbed macromolecules normalized by the surface area *L_x_* × *L_y_*. A macromolecule was considered to be adsorbed if at least one of its monomer units had coordinate *z* < 1.0. The values σ_mol_ were calculated as the total amount of the charged groups belonging to the adsorbed chains, multiplied by *S*_0_, and divided by the surface area. Subsequently, we have studied the peculiarities of the chains’ diffusion within the adsorbed layers.

One should note that our coarse-grained model with the implicit charge on the surface is a generalized model for a wide range of problems. Dealing with some particular problem and comparing the results with real experiment, one may need to take into account other factors such as the distances between the charged groups on the surface and their distribution, the interaction of monomer units with the surface, solvent quality, etc. [20,21].

## 3. Results and Discussion

### 3.1. Surface Overcharging

First, the systems consisting of chains with *N* = 48 monomer units were studied. The fraction of charged monomer units (ionization degree) ranged from *f* = 0.0625 to *f* = 1.0, and the value of the surface charge density ranged from σ_surf_ = 0.05 to σ_surf_ = 0.88.

**Figure 3:**
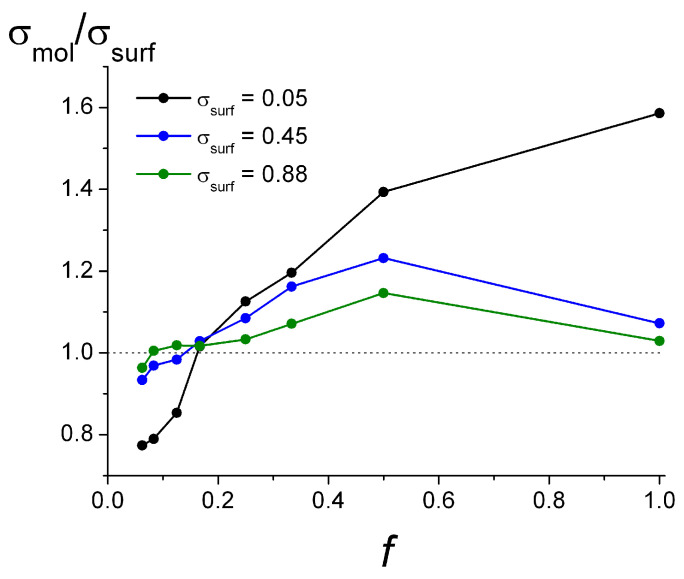
The relative density of the charged adsorbed monomer units (σ_mol_/σ_surf_) depending on ionization degree *f* at different surface charge σ_surf_.

In Figure 3 we present the overall charge density of the adsorbed monomer units σ_mol_ depending on the ionization degree *f*. The dependencies are given for different surface charge densities σ_surf_.

As it is seen, on the weakly charged surface (σ_surf_ = 0.05), the charge density monotonically grows from σ_mol_/σ_surf_ = 0.77 for the polyelectrolytes with *f* = 0.0625 to σ_mol_/σ_surf_ = 1.59 for *f* = 1. The complete charge of the adsorbed layer becomes larger than the surface charge (σ_mol_/σ_surf_ > 1.0) for the chains with *f* ≥ 0.17.

For the strongly charged surfaces, it is seen that the charge of the adsorbed macromolecules is high (σ_mol_/σ_surf_ ≈ 0.95) already at the smallest ionization degree, whereas the surface charge is slightly overcompensated for the chains with *f* = 0.17 and *f* = 1, and in the intermediate range of *f*, the total charge of the layer exceeds the surface charge. For the highest surface charge studied (σ_surf_ = 0.88), the curve is the closest to σ_mol_ = σ_surf_.

The results presented In Figure 3 demonstrate undercharging of the surface in all cases when the ionization degree is very low. That may be explained by entropic reasons: for the weakly charged chains, the entropy loss by transfer into an adsorbed state may play a more significant role than the energy gain of electrostatically induced adsorption. However, for some particular problems, this limiting case of low *f* may require consideration of the abovementioned factors, such as surface charge distribution.

It is worthwhile to mention that the number of charged groups in the close vicinity to the charged surface (at the distance *H_z_* ≤ 1.0) normalized to the total surface charge varied within the interval of 0.1 for *f* = 0.0625 on the weakly charged surface to 0.82 for *f* = 1 on the most highly charged surface. The rest of the charged monomer units in adsorbed macromolecules are distributed farther from the surface. Also, their total charge is partly compensated by counterions that are accumulated in the layer and that were not taken into account when calculating the layer charge; σ_mol_, presented in Figure 3, is the total number of charged monomer units. The analysis of the total layer charge, taking into account the counterions within the adsorbed layer, is presented in the Supplementary Materials (Figure S2).

### 3.2. Surface Coverage

**Figure 4:**
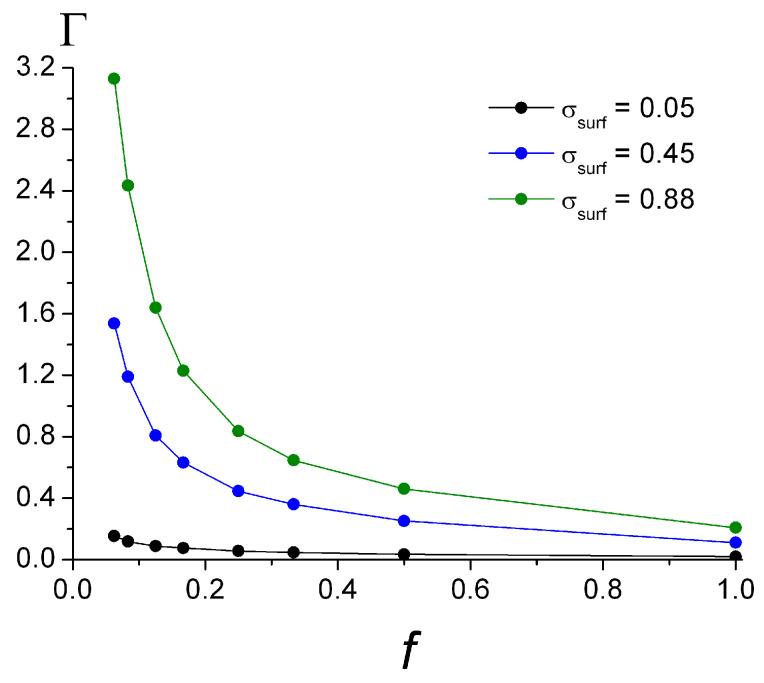
The surface coverage Γ depending on ionization degree *f* at different surface charge σ_surf_.

The surface coverage Γ depending on *f* is shown in Figure 4. With the growth of *f*, the total amount of the adsorbed monomer units per unit area Γ decreases abruptly at the small values of *f* and more slowly at high *f*. Comparing the curves, it is seen that the lower the ionization degree of the macromolecules, the more pronounced the growth of the surface coverage with the increase in σ_surf_. The dependencies can be approximated as Γ ~ *f* ^−0.72^ for σ_surf_ = 0.05 and are close to Γ ~ *f* ^−1^ for the high surface charges: Γ ~ *f* ^−0.92^ for σ_surf_ = 0.45 and Γ ~ *f* ^−0.96^ for σ_surf_ = 0.88 (see Figure S3 in Supplementary Materials).

### 3.3. Chains Conformations

The obtained results on the coverage and layer charge dependencies can be explained by the evaluation of the chains conformations within the adsorbed layers.

**Figure 5:**
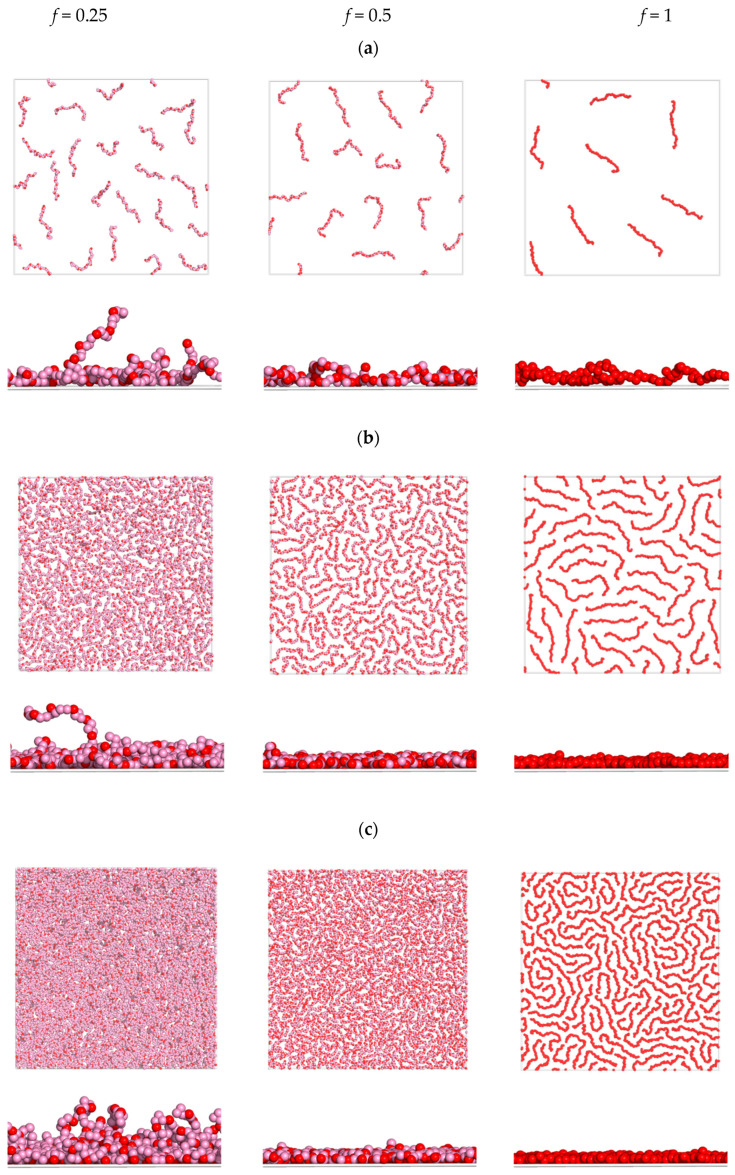
Snapshots of the top and side views of the simulation cell at σ_surf_ = 0.05 (**a**), σ_surf_ = 0.45 (**b**), and σ_surf_ = 0.88 (**c**) for different *f*. Counterions are not shown.

The snapshots demonstrating the conformations of the macromolecules for different *f* and σ_surf_ are shown in Figure 5.

The increase in the ionization degree *f* at the fixed surface charge σ_surf_ leads to the stiffening of the polyelectrolyte chains, their unfolding and flattening along the surface, and a visible decrease in the surface coverage.

At the low density of the surface charge (Figure 5a), the chains are freely distributed on the surface and do not overlap, whereas the side view demonstrates the formation of loops and tails. Visibly, the macromolecules on the weakly charged surface (Figure 5a) have the longest loops and tails, and for *f* = 0.25 and *f* = 0.5, the layers closest to the surface accommodate a large fraction of the neutral units along with the charged ones (see Figures S4 and S5 of Supplementary Materials). The loops are observed even for the case of fully charged chains with *f* = 1 (see Figure S5). The charged monomers in these loops repel each other, stretch the chains, and cause the chains to be evenly distributed over the surface.

The next snapshots, obtained with the growth of the surface charge, show the view of the densely covered surfaces (Figure 5b,c).

The macromolecules with a relatively low ionization degree (*f* = 0.25), with the increase in the surface charge, demonstrate the folding of the chains with the formation of more loops and tails. That explains the sharply increasing surface coverage Γ with the growth of σ_surf_ (seen in Figure 4 for *f* = 0.25). It is seen that with the increase in the surface charge density, the near-surface layer accommodates more charged units and displaces neutral units, indicating that more chains may be adsorbed due to their folding perpendicular to the surface and shifting along the surface (Figure S4). The ratio σ_mol_/σ_surf_ in this case undergoes some decrease with the growth of σ_surf_, which could be due to spatial restrictions (see Figure 3 for *f* = 0.25).

On the highly charged surfaces (Figure 5b,c), the polymers with high ionization degrees form a thin layer, where most units are located within a thin part of the adsorbed layer directly near the surface, and a small fraction of units forms a loose layer above it. The probability of the formation of loops and tails in this case significantly diminishes (Figure S5). At σ_surf_ = 0.45, the macromolecules with *f* = 0.5 are tightly packed on the surface and stretch locally along the surface, and with the highest degree of ionization (*f* = 1), the number of adsorbed macromolecules decreases, and their stretching increases markedly. At the higher surface charge (σ_surf_ = 0.88), the number of adsorbed macromolecules becomes larger and highly charged macromolecules (*f* = 1) adopt both totally stretched and folded conformations.

To quantify this effect, the mean components of the gyration radius along the surface $R_{xy}=\sqrt{(R_{x}^{2}+R_{y}^{2})/2}$ and perpendicular to the surface $R_{z}$ for the chains with *f* = 1 were evaluated. For σ_surf_ = 0.05, they are equal to $R_{xy}=6.7$ and $R_{z}=1.0$. For σ_surf_ = 0.45, $R_{z}$ becomes smaller, $R_{z}=0.6$, and $R_{xy}$ grows, $R_{xy}=7.2$. This means that the chains flatten perpendicular and stretch along the surface. With the further increase in the surface charge (σ_surf_ = 0.88), both components decrease, $R_{xy}=5.9$ and $R_{z}=0.3$. The macromolecules become almost two-dimensional and much less stretched.

### 3.4. Experimental Data [46]

In the experiment carried out in ref. [46], the adsorption of cationic copolymers on the surface of anionic borosilicate glass microspheres was studied. Copolymers with various content of cationic groups (*f*), and with either hydrophobic or hydrophilic (zwitterionic) neutral groups, were considered. The surface of the microspheres carried negatively charged silanol groups with the surface density of three groups per 1 nm^2^, which can be regarded as a high surface charge density in our computer simulation model. The efficiency of complexation between the cationic copolymers and glass microspheres was evaluated by measurement of the copolymers’ concentration in the supernatant, [CC]_free_, depending on the total concentration of the copolymers in the suspension, [CC]. With the [CC] increase, the value of [CC]_free_ was zero until [CC]_max_ of the maximum surface coverage was reached, and then [CC]_free_ grew linearly. We compare [CC]_max_ with the surface coverage Γ of our model. Accordingly, the product [CC^+^] = [CC]_max_ × *f* is compared with σ_mol_.

Experiments indicated that in hydrophobic copolymers, the amount of cationic groups in the saturated adsorption layers [CC^+^]_phob_ does not significantly depend on the fraction *f*, and [CC]_max_ is a decreasing function of *f*. However, [CC^+^]_phob_ values have a substantial spread: from about 20 × 10^4^ mol/L at the highest and lowest studied *f* = 0.24, *f* = 0.98 to 33 × 10^4^ mol/L at the intermediate *f* = 0.61. That is in good agreement with the results of the present simulations for the highly charged surfaces (Figure 3). In the hydrophilic copolymers, [CC^+^]_phil_ increases at low values of *f* and reaches a plateau at *f* ~ 0.2, while the surface coverage [CC]_max_(*f*) is the bell-shaped function growing at low *f* and decreasing at *f* > 0.2. The mechanism of the hydrophilic chains adsorption was explained in [46] based on the theory of the polyampholytes adsorption on charged particles [47], which argues that the adsorption efficiency is determined by the balance between the attraction of polymer chains to the surface induced by their polarization in the electric field of the particle and the repulsion between the groups of the polymer. Leaving aside the features of the adsorption curves at low *f* arising from the nature of the neutral groups, the dependencies of the surface coverage and the densities of the charged adsorbed groups for *f* > 0.2 obtained in the simulation adequately describe the experimental results.

### 3.5. Diffusion

With the competition between the increase in the adsorbed charged groups amount and the decrease in the total amount of the adsorbed groups, one can expect non-trivial behavior of the in-plane diffusion coefficient depending on the ionization degree *f*.

**Figure 6:**
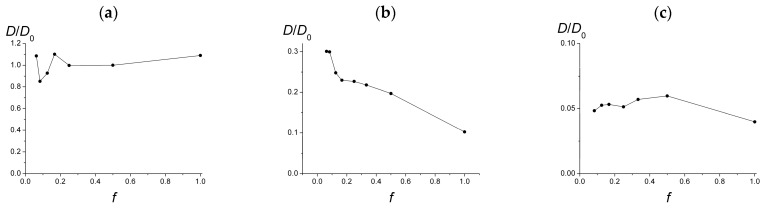
Lateral diffusion coefficient of the single chain at σ_surf_ = 0.05 (**a**) and of macromolecules in the adsorbed layer at σ_surf_ = 0.05 (**b**), σ_surf_ = 0.45 (**c**) depending on *f*. *D*_0_ is the diffusion coefficient of single macromolecules with *f* = 0.5 and σ_surf_ = 0.05.

The lateral diffusion coefficients *D* depending on *f* are presented in Figure 6 in the cases of a single chain on a weakly charged surface (σ_surf_ = 0.05, Figure 6a) and the surfaces of different charge under the conditions of their maximum possible coverage by adsorbed macromolecules (Figure 6b,c). For clarity, in Figure 6, the values of *D* are normalized to this value *D*_0_ for a single chain with *f* = 0.5.

For a single chain (Figure 6a), the dependency has a spread at low *f* which can be attributed to a significant error in determining the MSD slope, and as a whole demonstrates that the diffusion coefficient slightly, up to ten percent, changes with *f* at the given values of system parameters: low surface charge and low polymerization degree.

On the same weakly charged surface with σ_surf_ = 0.05, the diffusion coefficient of the macromolecules within the adsorbed layers is much lower: compare Figure 6a,b. At *f* ~ 0.1, the diffusion coefficient *D* is approximately one-third of the diffusion coefficient of a single chain, and it falls monotonically with increasing *f* to one-tenth of the diffusion coefficient of a single macromolecule. We attribute such a substantial diminish in the diffusion coefficient to the intermolecular interactions of the macromolecules, which affect their mutual positions and hinder diffusion. These intermolecular interactions have a long-range Coulomb character and grow along with the macromolecular charge. As a result, as *f* increases, the diffusion coefficient decreases, despite a noticeable fall in the surface coverage.

For highly charged surface (σ_surf_ = 0.45), the diffusion coefficient of the macromolecules is much, ten times, less than that for the weakly charged surface (Figure 6b,c). Note that now in both cases we consider surfaces covered with the maximum possible number of macromolecules. The highly charged surface adsorbs much more polymer chains, which overlap heavily, strongly influence each other, and restrict mutual diffusion. Interestingly, the diffusion coefficient does not vary significantly along with *f*. It can be explained by the simultaneous influence of opposite factors: a noticeable fall in the surface coverage and a significant growth of macromolecular charge, which, respectively, promote and hinder diffusion.

**Figure 7:**
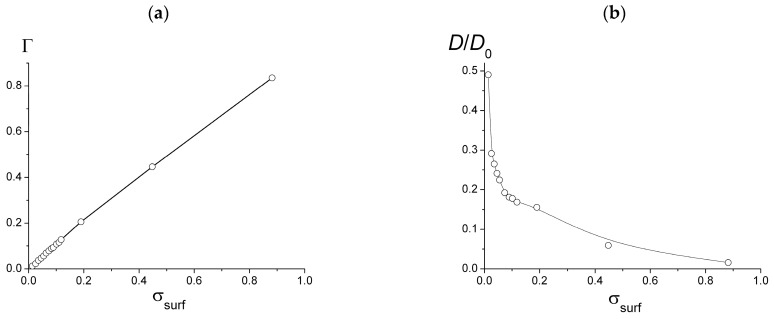
Dependence of the surface coverage (**a**) and lateral diffusion coefficient (**b**) on the surface charge density. *f* = 0.25.

The diffusion coefficient together with the surface coverage were measured for the chains with the a fixed ionization degree of *f* = 0.25 and different surface charge (Figure 7). While the analysis has shown that the surface coverage Γ linearly grows with σ_surf_ (Figure 7a), the diffusion coefficient steeply decreases in the region up to σ_surf_ ≈ 0.1 and then its decrease becomes much slower (Figure 7b). These regions correspond to the regime with the sparsely located macromolecules with fast diffusion (σ_surf_ < 0.1) and the regime of the crowded conditions with much slower diffusion (σ_surf_ > 0.1).

**Figure 8:**
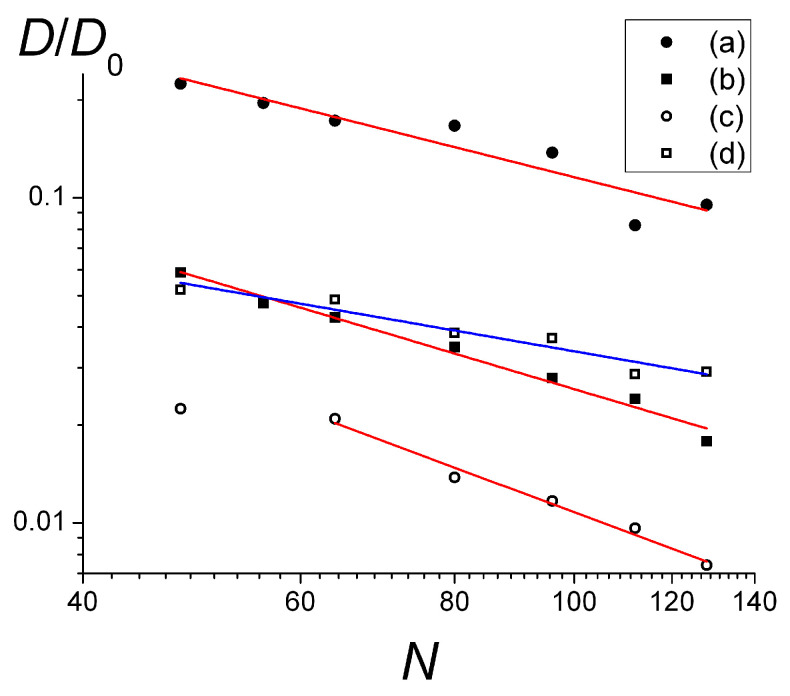
Dependence of lateral diffusion coefficient on the length of the chains. *f* = 0.25 and σ_surf_ = 0.05 (**a**), σ_surf_ = 0.45 (**b**); σ_surf_ = 0.88 (**c**) (approximated by the red lines); *f* = 1 and σ_surf_ = 0.45 (**d**) (approximated by the blue line).

The dependencies of the diffusion coefficient on the length of the chains *N* are shown in Figure 8a–c for weakly and strongly charged surfaces and *f* = 0.25. They were approximated as *D* ~ *N*^−0.95±0.16^ (Figure 8a), *D* ~ *N*^−1.13±0.06^ (Figure 8b), and *D* ~ *N*^−1.41±0.1^ (Figure 8c), respectively.

Thus, for the weakly charged surface and *f* = 0.25 (Figure 8a), the scaling dependence *D*(*N*) is pretty close to that predicted for the single macromolecules adsorbed on the smooth surface, following the Rouse (*D* ~ *N*^−1^) dynamics of non-entangled coils, which are not influenced by the neighboring chains [5,9,13].

For the stronger charged surface (Figure 8b), the scaling exponent is larger, the deviation from the Rouse law can be attributed to the fact that the chains in this case form a large number of loops and tails and obtain some degree of entanglement. The scaling dependence for the most highly charged surface (Figure 8c) with the thick adsorbed layer (see Figure 5c, *f* = 0.25) for the longer chains reveals an even higher value of the scaling exponent which is the closest to the evaluation obtained within the reptation theory framework in two dimensions (*D* ~ *N*^−3/2^).

Strongly charged macromolecules (*f* = 1) on the strongly charged surface demonstrate *D* ~ *N*^−0.66±0.09^ dependence (Figure 8d). It is seen that compared to the chains with a lower charge density (*f* = 0.25) on the same surface (Figure 8b), the diffusion of *f* = 1 chains is slower for shorter chains and faster for longer chains. The exponent in *D*(*N*) dependence is close to the −5/8 value derived for dense 2D polymer layers by Semenov et al. [11,12,13,14].

Here, we have investigated the structure of the adsorbed layers and distinguished several diffusion regimes for the adsorbed polyelectrolytes using the simple computer model. We present the tendencies that occur upon changing such parameters as the fraction of the charged monomer units, polymer length, and surface charge. We have considered the conditions of the maximal possible amount of polymer in the adsorbed layers and the rather high value of the Bjerrum length.

## 4. Conclusions

The adsorption of polyelectrolyte chains onto an oppositely charged surface and their lateral diffusion were investigated by means of a molecular dynamics simulation. It was shown that the surface density of the adsorbed chains Γ (surface coverage) depends on the ionization degree *f* as Γ ~ 1/*f* ^3/4^ for weakly charged surfaces and is close to Γ ~ 1/*f* for highly charged surfaces. The macromolecules are located more sparsely and acquire more elongated conformations with the increase in *f*.

The surface density of the charged units within the adsorbed layer σ_mol_ monotonically grows with *f* on the weakly charged surfaces and exceeds the surface charge density σ_surf_ at *f* > 0.2. For highly charged surfaces, σ_mol_ first grows until about *f* ≈ 0.25, then slightly exceeds σ_surf_ and becomes equal to it again at *f* = 1. This phenomenon is explained by the appearance of the “tails” and loops formed by the chains and bearing additional charge which, in part, is compensated by counterions.

The obtained dependencies on the highly charged surfaces are in line with the experimental results [46], demonstrating the significant decrease in the surface coverage with *f* and the existence of the spread in the amount of the adsorbed charged groups and its growth at the intermediate *f*.

Computer simulations have shown that the lateral diffusion coefficient of the chains *D* decreases with *f* on the weakly charged surfaces; while the surface coverage falls with *f*, the long-range Coulomb interactions slow down the diffusion.

On the highly charged surfaces with high surface coverage, *D* weakly depends on the ionization degree *f*. This result is due to the fact that the increase in the ionization degree, on one hand, leads to the decrease in the surface coverage and, on the other hand, leads to the growth of interchain repulsion.

With the increase in the surface charge density, the surface coverage linearly grows, while *D* first sharply decreases and then the decrease becomes much slower. That corresponds to the two regimes: the very mobile sparsely located macromolecules and the crowded conditions with slow diffusion.

The scaling *D*(*N*) depends on the surface and macromolecular charge. Different scaling laws were established: Rouse-type diffusion (*D* ~ *N*^−1^) for sparsely covered surfaces; ameboid-like diffusion (*D* ~ *N*^−5/8^) for dense 2D monolayer; and *D* ~ *N*^−γ^ (γ > 1) for systems with a large surface coverage and with visible numbers of loops and tails.

Thereby, we have first determined the relationships between the ionization degree of the polyelectrolytes, adsorbed on the oppositely charged surface, the surface coverage (and the amount of the charged groups in the adsorbed layers), and the mobility of the chains. The high charge density and high diffusivity of the adsorbed polyelectrolyte chains can be beneficial factors for practical applications of the surface coatings. For instance, these factors can play a major role in the ability of the coatings to efficiently trap bacteria, viruses, and other charged contaminant particles; the ability of the polymer chains in such coatings to inter-diffuse and heal damages; the response to the outer conditions, allowing one to remove the coatings, etc.

## Data Availability

The data presented in this study are available on request from the corresponding authors.

## Supplementary Materials

The following supporting information can be downloaded at: https://www.mdpi.com/article/10.3390/polym15132845/s1, Figure S1: An exemplary plot of mean-square displacement of the macromolecules vs time, linear and log scales. N = 48, f = 0.33, σ_surf_ = 0.05; Figure S2: The relative density of the charged adsorbed monomer units σmol and the opposite charge of the surface and counterions σ_surf_ + σ_c_; Figure S3: The surface coverage Γ depending on ionization degree f at different surface charge σ_surf_: the log-log plot and the slopes of the linear fit; Figure S4: The distribution of neutral and charged monomer units along the *z*-axis. *H_z_* is the distance from the surface. *f* = 0.25; Figure S5: The distribution of loops length *P*(*N_l_*) (a) and tails length *P*(*N_t_*) (b) for different *f* and σ_surf_.
