# Supplementary material for: Computer Simulation Insight into the Adsorption and Diffusion of Polyelectrolytes on Oppositely Charged Surface
Source: Polymers (Basel). 2023 Jun 28;15(13):2845. doi: 10.3390/polym15132845 (PMC10346556; doi:10.3390/polym15132845)
Supplement: Supplementary file 1 [file polymers-15-02845-s001.zip › polymers-2433471-supplementary.pdf]

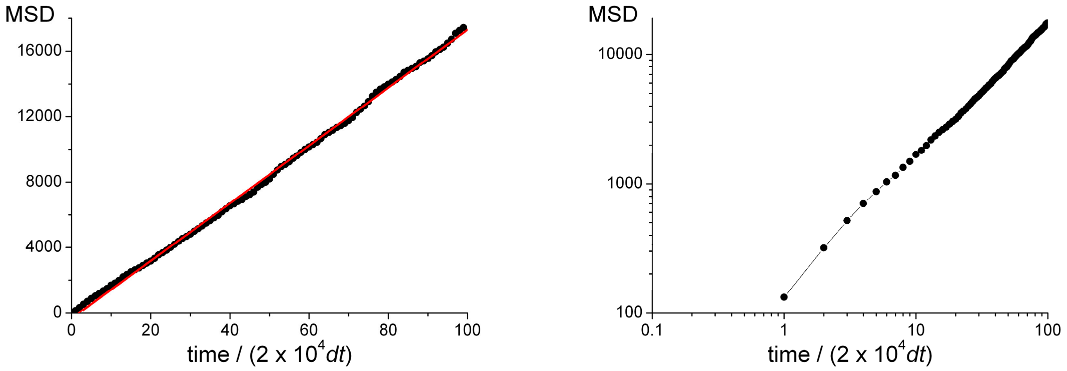

Figure S1. An exemplary plot of mean-square displacement of the macromolecules vs time, linear and log scales. $N = 48$, $f = 0.33$, $\sigma_{surf} = 0.05$.

The slope of the linear approximation (red line) determines the diffusion coefficient D (is equal to $4D$).

The standard deviation of the values of D is within 10%.

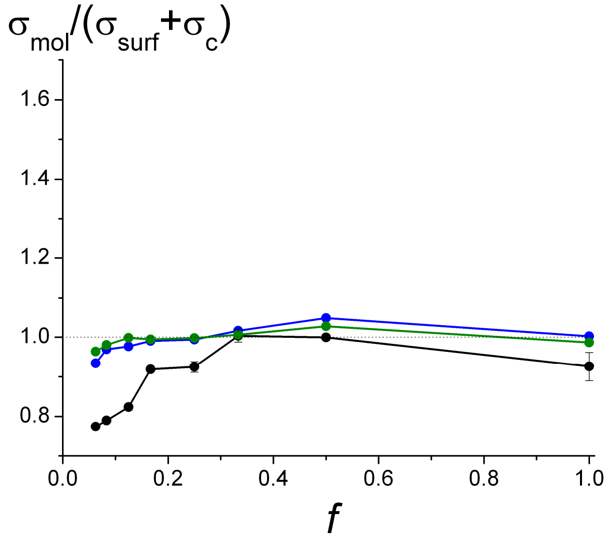

Figure S2. The relative density of the charged adsorbed monomer units σ_{mol} and the opposite charge of the surface and counterions $\sigma_{surf} + \sigma_c$.

The ratio of $\sigma_{mol}/(\sigma_{surf} + \sigma_c)$ was calculated as follows:

$\sigma_{mol}/(\sigma_{surf} + \sigma_c) = Q_{mol}/(Q_{surf} + Q_c)$,

where $Q_{mol} = \Gamma \times f$ is the number of charged monomer units within the adsorbed layer;

Q_c is the number of counterions within the adsorbed layer, their *z*-axis coordinate perpendicular to the surface does not exceed the maximal *z*-axis coordinate of the adsorbed polyelectrolyte monomer units;

$Q_{surf} = F_z \times L_x \times L_y/(2\pi \times l_B) - Q_{up}$.

The dependencies with and without taking into account the counterions of the macromolecules reveal the same tendency for surface undercharging for weakly charged polyelectrolytes.

On the strongly charged surfaces, taking into account the counterions diminishes the amplitude of the overcharging effect.

On weakly charged surface, the excess charge of adsorbed macromolecules is compensated by their counterions.

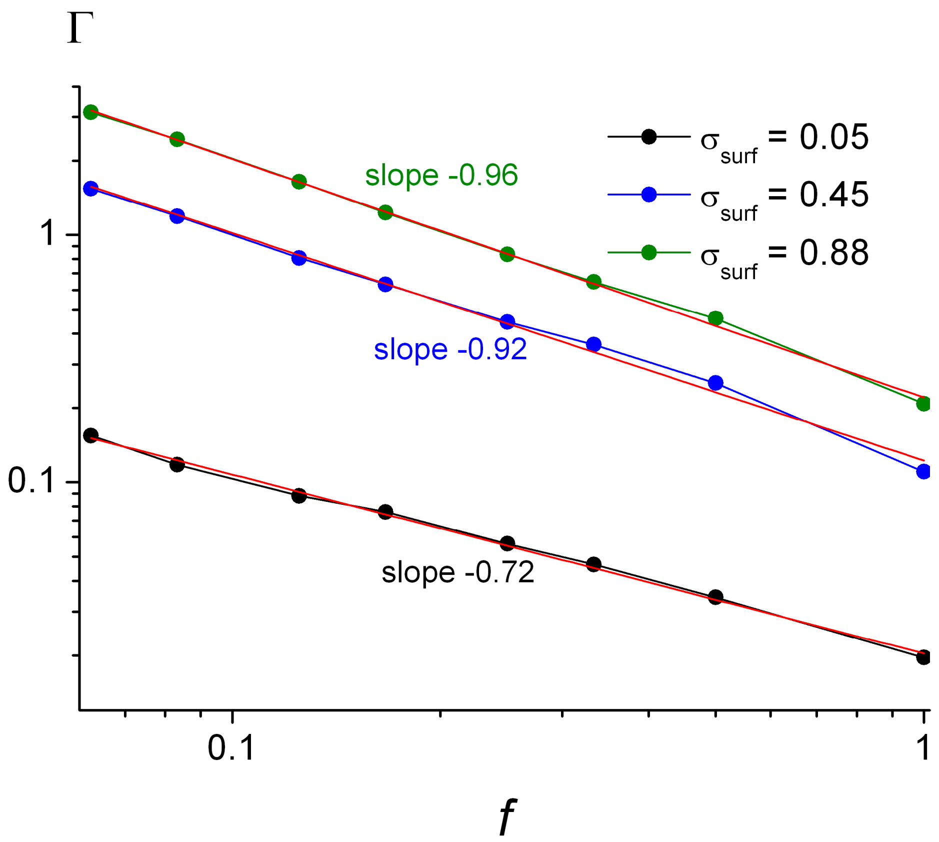

Figure S3. The surface coverage Γ depending on ionization degree *f* at different surface charge σ_{surf}: the log-log plot and the slopes of the linear fit.

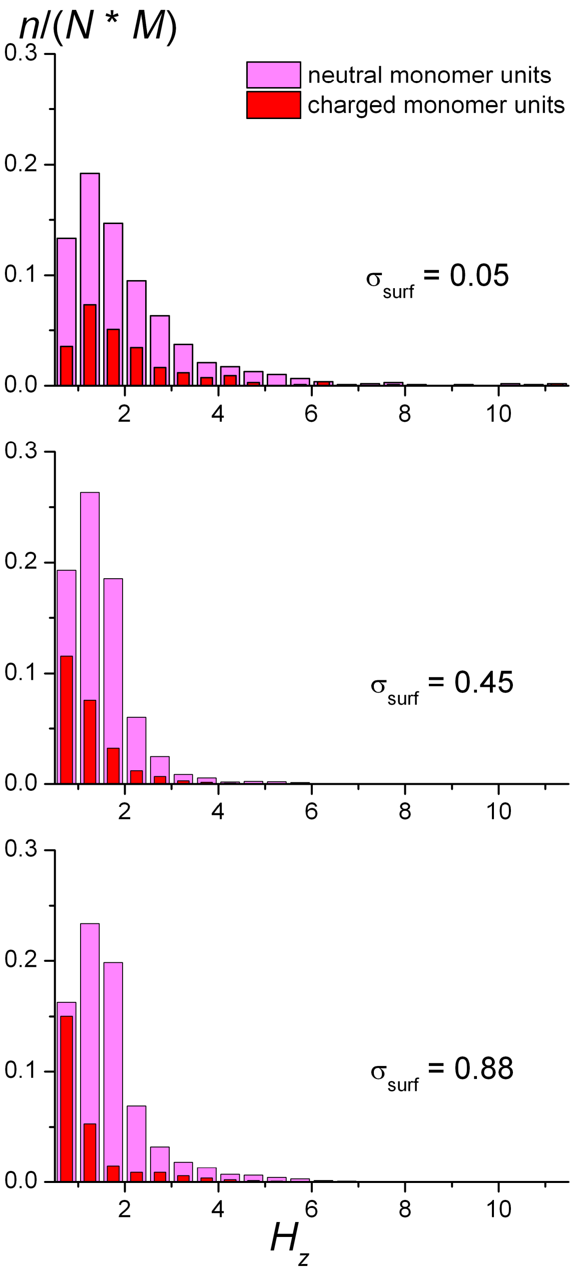

Figure S4. The distribution of neutral and charged monomer units along the *z*-axis. H_z is the distance from the surface. $f = 0.25$.

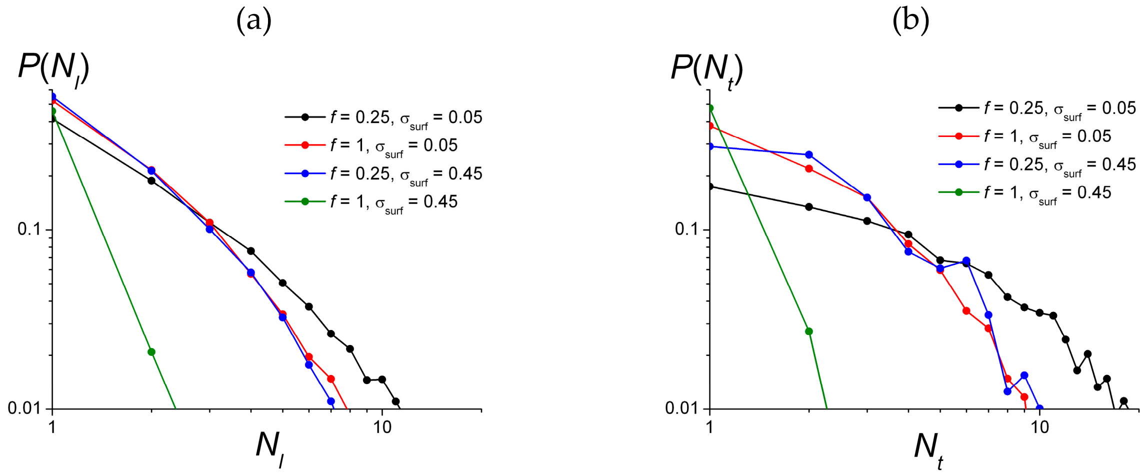

Figure S5. The distribution of loops length $P(N_l)$ (a) and tails length $P(N_t)$ (b) for different f and σ_{surf}.

The loop is defined as a chain fragment having both end-groups within the adsorbed layer and consisting of N_l consecutive monomer units, which are located at $H_z > 2$. H_z is the distance from the surface.

Tails are the ending fragments of the chains having one end in the adsorbed layer and consisting of N_t consecutive monomer units, which are located at $H_z > 2$.

The probability $P(N_l)$ (or $P(N_t)$) is the fraction of the loops (or tails) of the given length among all the loops (tails).
